# Inter-observer variations of the tumor bed delineation for patients after breast conserving surgery in preoperative magnetic resonance and computed tomography scan fusion

**DOI:** 10.1186/s12885-021-08546-5

**Published:** 2021-07-20

**Authors:** Jie Jiang, Jinhu Chen, Wanhu Li, Yongqing Li, Yiru Chen, Zicheng Zhang, Chengxin Liu, Dan Han, Hongfu Sun, Baosheng Li, Wei Huang

**Affiliations:** 1 Weifang Medical University, Weifang, China; 2 Shandong Cancer Hospital and Institute, Shandong First Medical University and Shandong Academy of Medical Sciences, 440 Jiyan Road, Jinan, 250117 Shandong Province China; 3 Department of Radiation Oncology, Shenzhen Traditional Chinese Medicine Hospital, The Fourth Clinical Medical College of Guangzhou University of Chinese Medicine, Shenzhen, China

**Keywords:** Breast cancer, Radiotherapy, Tumor bed, Magnetic resonance imaging, Computed tomography

## Abstract

**Purpose:**

Tumor bed (TB) delineation based on preoperative magnetic resonance imaging (pre-MRI) fused with postoperative computed tomography (post-CT) were compared to post-CT only to define pre-MRI may aid in improving the accuracy of delineation.

**Methods and materials:**

The pre-MRI imaging of 10 patients underwent radiotherapy (RT) after breast conserving surgery (BCS) were reviewed. Post-CT scans were acquired in the same prone position as pre-MRI. Pre-MRI and post-CT automatically match and then manual alignment was given to enhance fusion consistency. Three radiation oncologists and 2 radiologists delineated the clinical target volume (CTV) for CT-based. The gross target volume (GTV) of pre-MRI-based was determined by the volume of tumor acquired with 6 sequences: T1, T2, T2W-SPAIR, DWI, dyn-eTHRIVE and sdyn-eTHRIVE, expended 10 mm to form the CTV-pre-MRI. Planning target volume (PTV) for each sequence was determined by CTV extended 15 mm, trimmed to 3 mm from skin and the breast-chest wall interface. The variability of the TB delineation were developed as follows: the mean volume, conformity index (CI) and dice coefficient (DC).

**Results:**

The mean volumes of CTV and PTV delineated with CT were all larger than those with pre-MRI. The lower inter-observer variability was observed from PTV, especially in sdyn-eTHRIVE in all sequences. For each sequence of pre-MRI, all DCs were larger than post-CT, and the largest DC was observed by sdyn-eTHRIVE sequence fusion to post-CT. The overlap for PTV was significantly improved in the pre-MRI-based compared with the CT-based.

**Conclusions:**

TB volumes based on pre-MRI were smaller than post-CT with CVS increased. Pre-MRI provided a more precise definition of the TB with observers performed a smaller inter-observer variability than CT. Pre-MRI, especially in sdyn-eTHRIVE sequence, should help in reducing treatment volumes with the improved accuracy of TB delineation of adjuvant RT of breast cancer.

## Background

Radiotherapy (RT) is an indispensable treatment for many patients with early breast cancer after breast-conserving surgery (BCS) [1]. Despite the existence of various RT techniques, tumor bed (TB) boost after whole breast irradiation or accelerated partial-breast irradiation stands out because of its advantage of reducing the irradiated area with a low rate of local recurrence [2]. Identification and contouring TB volume as accurately as possible could reduce recurrence and improve cosmetic effect [3].

A few previous studies have reported that there are significant variations in defining target volumes for breast RT [4]. For example, Hurkmans et al. [5] observed that the breast volume based on computed tomography (CT) imaging delineated by different observers varied by 17.5%. A consensus on the target volume is lacking on the delineation of the target volume. The TB delineation is based on the surgical cavity formed after BCS of breast cancer. A few clinical researches have been developed to improve the delineation of the TB, including the use of seroma cavity, preoperative notes, clinical palpation, surgical clips, surgical scar and ultrasound(US)/CTimaging/mammography [6]. However, these methods are not perfect. The seroma cavity and the clip positions change significantly over days or weeks postoperatively, which are non-negligible factors affecting the delineation of the TB volume [7]. CT is a conventional method used for breast delineation. Distinguishing TB from normal glandular breast tissue is not satisfactory with the use of postoperative CT (post-CT) alone [8, 9]. Thus, some studies explored an approach involving the co-registration between CT and other breast imaging examinations, such as US, mammography and magnetic resonance imaging (MRI). MRI was more clinically accurate in tumor size estimation than mammography and US in 30% of breast cancer patients [10]. Moreover, MRI, which has superb soft-tissue contrast, could improve observer concordance, reproducibility and anatomic accuracy compared with CT [11, 12]. A previous study assessed the inter-observer variability of surgical bed delineation after BCS pointed out that the fusion of CT and MRI should be used for surgical bed delineation [8]. This study aimed to evaluate the reproducibility of TB delineation and localisation based on prone pre-MRI and post-CT imaging fusion. It also aimed to compare the inter-observer variability between post-CT and pre-MRI.

## Methods

### Clinical characteristics of the patients

Ten patients with T1N(0–1)M0 breast cancer who underwent lumpectomy and lymph node dissection between June 2016 and June 2019 were enrolled in this study. Patients scheduled to adjuvant radiotherapy were pathologically diagnosed with invasive ductal carcinoma with 3–10titanium clips placed round the lumpectomy cavity during surgery. The study exclusion criteria were as follows: patients received endocrinotherapy with oncoplastic surgery, neoadjuvant chemotherapy and harboring contraindication. Patient characteristics are displayed in Table 1.

**Table 1:** Clinical characteristics of the included 10 patients

Patients’ characteristics	NO	%
Age median		
≤ 40	3	30
40–50	3	30
50–60	4	40
Location		
Left	7	70
Right	3	30
Pathologic T stage		
T1b	1	10
T1c	9	90
Histologic grade		
I	8	80
II	2	20
Pathological type		
Invasive ductal carcinoma	10	100
Days from MRI to surgery(median, range)	1,1–4	100
Days from surgery to simulation(median, range)	102,26–173	100
CVS		
1	0	
2	1	10
3	5	50
4	4	40
5	0	

### Pre-MRI and post-CT simulation

The pre-MRI simulation of enrolled patients usually was performed within 1 week before BCS. Furthermore, the process of pre-MRI imaging was performed using Philips Achieva 3.0 T (60 cm bore diameter) in the routine prone position. Six sequences of pre-MRI were included for each patient, namely, T1, T2, T2W-spectral presaturation attenuated inversion-recovery (SPAIR), DWI, dynamic-enhanced T1 high-resolution isotropic volume excitation (dyn-eTHRIVE) and subtraction of dynamic-enhanced T1 high-resolution isotropic volume excitation (sdyn-eTHRIVE). The post-CT data were acquired for the shortest month after BCS and up to 6 months with postoperative adjuvant chemotherapy. Moreover, post-CT simulation required the same prone position as pre-MRI but with the use of a specific immobilisation device, namely, a Philips large-bore CT scanner (HighSpeed, GE). The post-CT images were obtained at 3 mm slice thickness, but the slice interval of T1, T2, T2W-SPAIR and DWI was 4 mm. The dyn-eTHRIVE and sdyn-eTHRIVE were collected at 1 mm slice thickness. To keep the slice thickness consistent, CT, dyn-eTHRIVE and sdyn-eTHRIVE images were reconstructed to 4 mm slice thickness. The echo times for T1, T2, T2W-SPAIR and DWI were 10 ms, 120 ms, 60 ms and 51 ms, and repetition times were 495 ms, 4213 ms, 4600 ms and 7099 ms, respectively. For the acquisition of dyn-eTHRIVE, gadopentetate dimeglumine was administered intravenously at a dose of 0.1 mmol/kg and at a rate of 3 ml/s. Thus, eight sequences of dyn-eTHRIVE were composed of the first sequence before injecting the contrast-enhancing agent, and seven sequences of uninterrupted scanning were performed after the injection of the contrast agent. Sdyn-eTHRIVE included four sequences originating from the subtraction images of the dyn-eTHRIVE image. In collaboration with radiologists, we selected the sequence with superior visibility as the representative image of the dyn-eTHRIVE sequence and sdyn-eTHRIVE sequences. The DWI image with b = 800 s/mm2 was chosen as the image for delineation.

### Image co-registration

Both pre-MR and post-CT images were imported in the Eclipses’ Treatment Planning Systems for registration and contouring. Each pre-MRI sequence (T1, T2, T2W-SPAIR, DWI, dyn-eTHRIVE and sdyn-eTHRIVE) was individually registered with post-CT by rigid registration based on anatomy, i.e., the nipple, the tip of scapula and the sternum, especially glandular breast tissue. Manual alignment was performed to enhance fusion consistency. The focus was on the glandular breast tissue concordance. The image registration was accepted when deemed satisfactory by five observers.

### Structure delineation

**Fig. 1:**
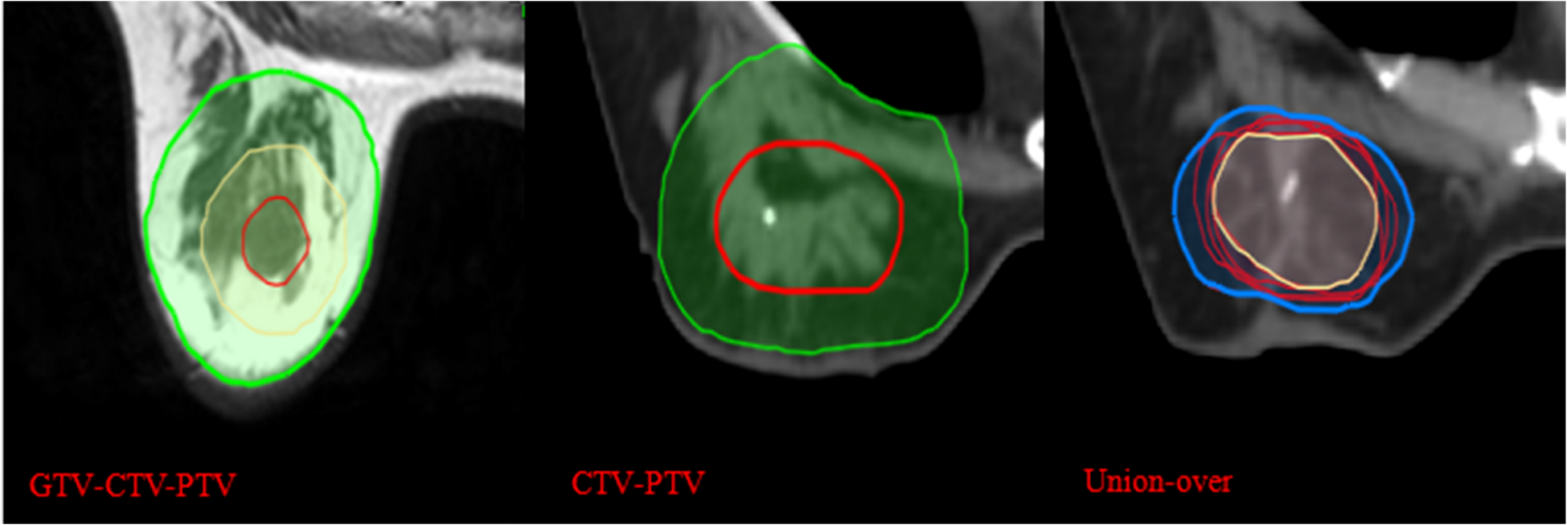
The delineation of target volume based on pre-MRI and post-CT drawn by one radiation oncologist, and the common and encompassing volumes of CTV drawn by 5 observers

**Fig. 2:**
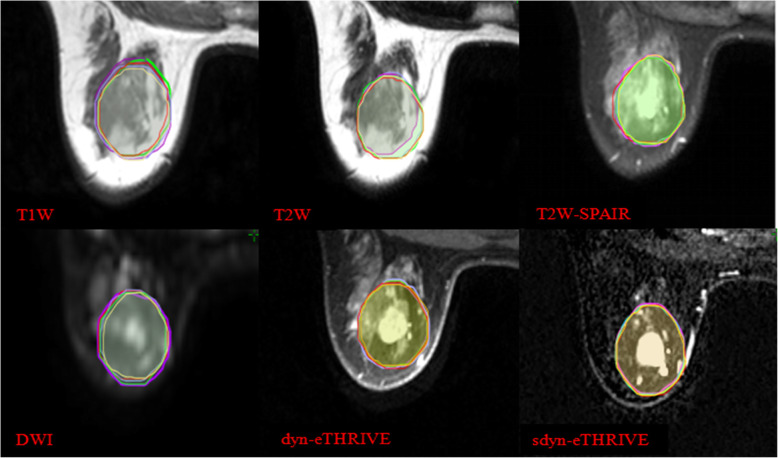
Delineations of CTV defined by 3 radiation oncologists and 2 radiologists in 6 pre-MRI sequences

This study only involved image processing, not human body research. Three radiation oncologists and two radiologists with much experience in breast cancer treatment participated in the delineation of the target volume. Clips were placed around the lumpectomy cavity. Before the study, all observers had no access to the medical records of all patients and contours of other observers. All observers did not undergo training for a consistent standard before delineation. One of authors ensured that guidelines were met, and coded information was saved. All observers were assigned to a cavity visualisation score (CVS) of 1–5 for each patient’s image before contouring the TB volume. The CVS is based on the guidelines of Smitt et al. [13], as follows: CVS-1 cavity not visualised; CVS-2 cavity was visualised with indistinct margins; CVS-3 cavity was visualised with some distinct margins and heterogeneous appearance on CT; CVS-4 cavity with mild heterogeneity with distinct margins on CT; and CVS-5 homogenous appearance of the cavity, and all margins were clearly seen on CT. The breast window of post-CT was first presented for contouring, whereas contouring on a modality was not allowed to refer to another modality. Every observer determined the clinical target volume (CTV-CT) of TB on post-CT image according to clinical experience combined with other supplementary methods, such as seroma, clips and other marks for clinical application. The definition of the planning target volume (PTV-CT) was CTV-CT extended to 15 mm by the planning system. For pre-MRI, images of six sequences (T1, T2, T2W-SPAIR, DWI, dyn-eTHRIVE and sdyn-eTHRIVE) were provided to contour the gross tumor volume (GTV) by the contrast difference between the tumor tissue and the surrounding normal tissue. Post-CT images of the same patient should be hidden when contouring. No clinical history or localisation information (such as US location of tumor) was provided. The CTV-MRI was created with a 10 mm geometrical extension of the GTV based on the system tools. A 10 mm microscopically tumor free margin gives the best positive predictive value based on pathology [14]. The surgeon expanded 10 mm around the tumor after BCS to avoid residual lesions. Then, a 15 mm extension of CTV-MRI was used to define the PTV-MRI. In all PTV, restrictions limited to 3 mm from the skin and to the breast-chest wall interface were met. The details of delineation are shown in Figs. 1 and 2.

### Statistical analysis

The CTV and PTV were scaled out by the geometrical expansion of seroma target volume or primary tumor volume followed by trimming. The mean volume and conformity index (CI) were compared between the radiation oncologists for each patient to determine inter-observer variability. The ratios reflected the consistency of the observers and were used to calculate the overlapping volume and the union volume for each patient. A dice coefficient (DC) indicated perfect concordance with the increase. The value of DC was calculated using the ratio of overlapping volume and the average volume contoured by five observers. Analysis of variance of one-way was used to compare all sequences. A value of P less than 0.05 was considered significant.

## Results

**Fig. 3:**
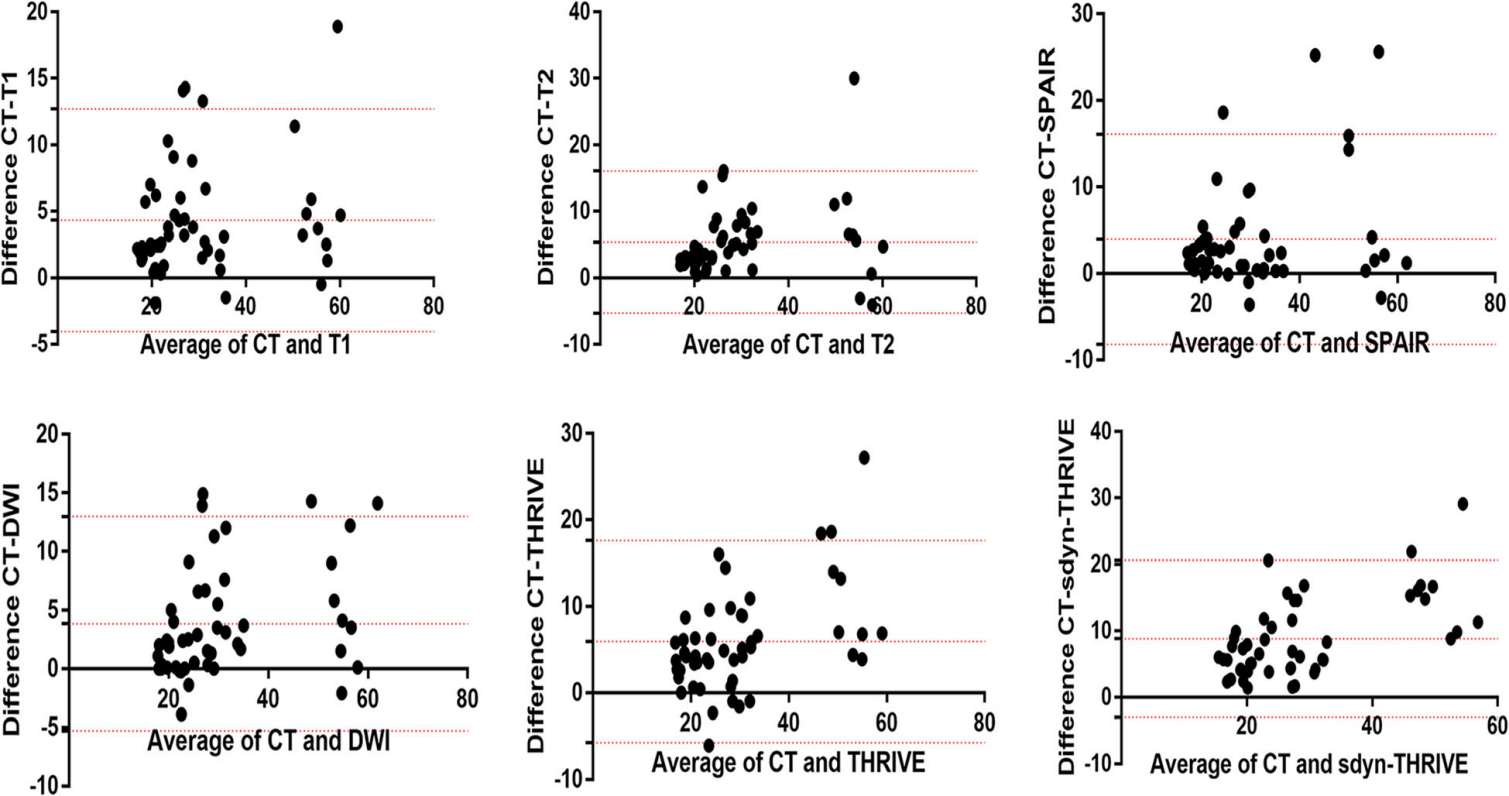
Bland-Altman agreement plots. Consistency of breast volume were performed by the mean breast volume for each pair of image techniques, with each point representing CTV of one observer. The upper line indicate the upper 95% limits of agreement. The middle line indicate the mean difference. The lower line indicate the lower 95% limits of agreement. The smaller the range between upper line and lower line is, the better agreement is

**Fig. 4:**
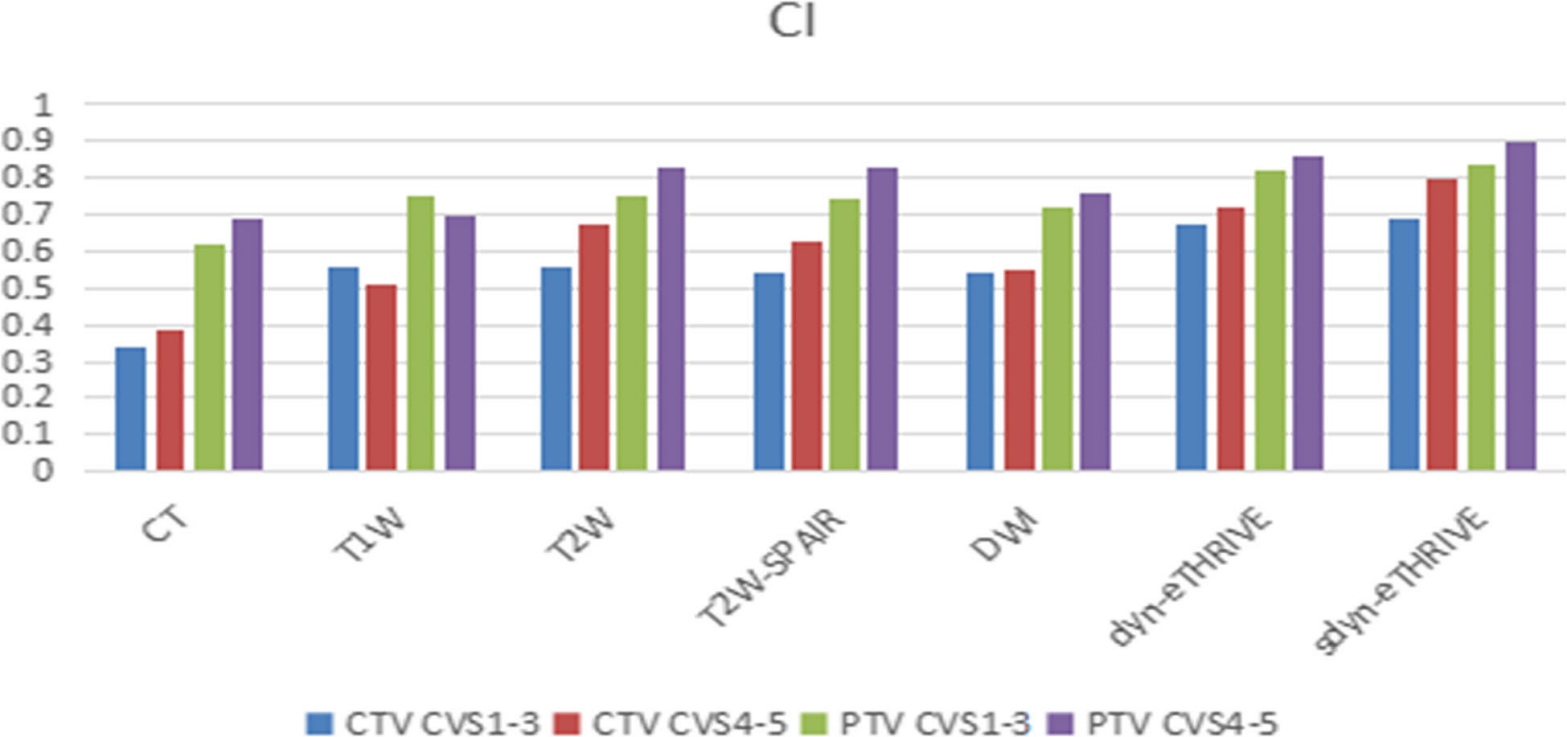
The observers variety of CTV and PTV in CVS scored 1–3 compared scored 4–5

As shown in the Tables 2 and 3, for the different patients, the volumes delineated by five observers on CT alone or CT-pre-MRI images were compared. The mean volume ± SD of the CTV with CT-based delineation was 33.1 ± 14.09. The mean volumes±SD delineated with CTV-pre-MRI in T1, T2, SPAIR, DWI, dyn-eTHRIVE and sdyn-eTHRIVE were 28.76 ± 13.49, 27.73 ± 13.06, 29.16 ± 12.51, 29.25 ± 12.59, 27.15 ± 11.33 and 24.28 ± 10.63, respectively, indicating significant differences between CTV-CT and CTV-pre-MRI among the six sequences (*P* = 1.5 × 10^− 5^, *P* = 4 × 10^− 6^, *P* = 2.5699 × 10^− 2^, *P* = 5.98 × 10^− 4^, *P* = 3.611 × 10^− 3^, *P* = 2.72 × 10^− 4^). Similarly, the mean volume of PTV-CT was 194.51 ± 53.11, which was larger than that of any other sequence for PTV-pre-MRI (*P* = 1.7 × 10^− 5^). The mean percentages of target volume reduction in CTV-pre-MRIs from CTV-CT were 14.19, 17.19, 11.76, 11.45, 17.4 and 26.76% and those in PTV-pre-MRIs were 24, 26, 23, 21, 26 and 30% for T1, T2, SPAIR, DWI, dyn-eTHRIVE and sdyn-eTHRIVE, respectively. Significant differences were observed in the mean volume reductions delineated by the five observers on sdyn-eTHRIVE sequence. Compared with the DC-CTV values of 0.56, 0.69, 0.75, 0.74, 0.72, 0.82 and 0.86 for post-CT, T1, T2, T2W-SPAIR, DWI, dyn-eTHRIVE and sdyn-eTHRIVE, the values were increased to 0.79, 0.84, 0.88, 0.88, 0.86, 0.91 and 0.93 for DC-PTV, respectively. The CI is the volume percentage on which all observers agree on each modality either in CT-based or MRI-based delineations. For CTV, CIs drawn by all observers for each patient were 0.37, 0.54, 0.6, 0.58, 0.55, 0.69 and 0.74 in CT, T1, T2, SPAIR, DWI, dyn-eTHRIVE and sdyn-eTHRIVE, respectively. Meanwhile, the CIs of PTV for all sequences were calculated as 0.65, 0.73, 0.79, 0.78, 0.74, 0.84 and 0.87, indicating that lower inter-observer variability was observed from PTV, especially in the sdyn-eTHRIVE sequence. Pairwise comparisons showed a highly significant difference between CT and MRI scores in T1, T2, SPAIR, DWI, dyn-eTHRIVE and sdyn-THRIVE. The delineation with sdyn-THRIVE was significantly consistent compared with any other pre-MRI sequence fused on post-CT. According to the consistency of CTV between the CT and each MRI sequence in the Bland-Altman agreement plots shown in the Fig. 3, the smaller range of confidence interval appeared in the T2 and sdyn-THRIVE sequence. The mean volume for the CTVs with CVS of 1–3 and 4–5 were 35.3 and 27.93, with CIs of 0.36 and 0.38, respectively. The observers variety of CTV and PTV in CVS scored 1–3 compared scored 4–5 shown in the Fig. 4.

**Table 2:** Mean volumes of delineation between post-CT and pre-MRI of 6 sequences

Volume	CTV(mean ± SD)	*P*^*2*^	PTV (mean ± SD)	*P*^*2*^
CT	33.1 ± 14.09		194.51 ± 53.11	
T1W	28.76 ± 13.49	1.5 × 10^−5^	150.27 ± 54.47	2 × 10^−6^
T2W	27.73 ± 13.06	4 × 10^−6^	146.3 ± 49.33	1 × 10^−6^
T2-SPAIR	29.16 ± 12.51	2.5699 × 10^−2^	150.68 ± 47.19	1.12 × 10^−4^
DWI	29.25 ± 12.59	5.98 × 10^−4^	154.57 ± 45.59	7 × 10^−6^
dyn-eTHRIVE	27.15 ± 11.33	3.611 × 10^−3^	144.28 ± 41.83	4.9 × 10^−5^
sdyn-eTHRIVE	24.28 ± 10.63	2.72 × 10^−4^	136.04 ± 38.62	1.7 × 10^−5^
F	35.764		152.695	
*P*^*1*^	8.041 × 10^−11^		6.6615 × 10^−18^	

*P* value^1^ were calculated by two-way analysis of Scheirer-Ray-Hare

*P* value^2^ were calculated by paird-samples T test to assess the difference between post-CT and pre-MRI sequences

**Table 3:** The reduce rates of mean volumes and observers consistency for CTV and PTV in CT-based compared with pre-MRI-based of 6 sequences

	CT	T1W	T2W	T2W-SPAIR	DWI	dyn-eTHRIVE	sdyn-eTHRIVE	F	*P*
CTV volume reduce%		14.19%	17.19%	11.76%	11.45%	17.4%	26.76%		
DC	0.56	0.69	0.75	0.74	0.72	0.82	0.86	7.406	5 × 10^−6^
CI	0.37	0.54	0.6	0.58	0.55	0.69	0.74	10.076	8.7121 × 10^−8^
PTV volume reduce%		24%	26%	23%	21%	26%	30%		
DC	0.79	0.84	0.88	0.88	0.86	0.91	0.93	4.621	1 × 10^−3^
CI	0.65	0.73	0.79	0.78	0.74	0.84	0.87	6.188	3.18 × 10^−5^

*P* value were calculated by one-way analysis of ANOVA to compare the differences of consistency parameters among observers

## Discussion

In most studies on breast RT, the definition of target volume is the weakest point in the chain of treatment processes. Regardless of tumor size and location, breast density, pathologic resection volume and interval from BCS to RT have a certain influence on the seroma cavity [15]. As a routine examination of breast, US has limited ability to define the boundary of tumor in RT. It is difficult for US to form certain criteria for delineating the target volume, but it may usefully support CT in defining the cranial and posterior extensions, especially when tumors are localized there [16]. Postoperative MRI provides a precise lumpectomy cavity delineation modality, as reported by a previous research on the improvement of the accuracy of target delineation [17]. The present research showed that the lumpectomy cavity based on the union of MRI sequences, T1, T2, TI inversion recovery, and dynamic contrast-enhanced MRI, especially on TI inversion recovery sequence, can accurately express the boundaries of the seroma cavity compared with CT only [6]. However, postoperative change still causes instability of the seroma and leads to the possibility of the clip transferring from place to place. The pre-MRI approach is widely used to determine the extent of the tumor and regional nodes and improves the further diagnosis of early breast cancer patients. The pre-MRI is superior to US and CT in determining the size and extension of the tumor. Thus, it can more accurately define the TB for breast cancer and reduce inter-observer variations [18–20]. In this study, the target volume and inter-observer consistency of pre-MRI-based delineation were better compared with those of CT-based delineation. Inter-observer variation was considered for T1, T2, T2W-SPAIR, DWI, dyn-eTHRIVE and sdyn-eTHRIVE images and was found to be reduced in post-CT for the CTV. Regardless of the sequence, the PTV trimmed to 3 mm from skin and to the breast-chest wall interface showed a lower difference than CTV. The improvement of contouring consistency was reflected in contouring tools and guidelines. The observers likely delineate target volume based on clinical experience. In addition, observing the factors that cause variability may be subject to differences in opinion considering target volume boundaries, uncertain approaches for incorporating treatment set-up and dosimetric limitations [4]. Thus, the achievement of high concordance among observers may be achieved if a protocol exists to maintain consistency in the delineation of breast target volume. Low inter-observer variability was obtained from the delineation of TB with high CVS in all sequences. Multiple factors, such as differences across individuals or infection after surgery, obscured any correlation between the lengths of time from breast conserving surgery to radiation therapy and the CVS. These correlations cannot be differentiated. Pre-MRI and post-CT imaging were performed in the prone position. Aside from increasing the level of inhomogeneity, patients with large breasts or large pendulous tissues may acquire acute skin reactions in the supine position. The prone position enhances dose distribution for target volume and optimises the sparing of the organ at risk [21]. Poor repeatability of the spatial position of the breast in each RT can greatly be alleviated by performing the imaging in the prone position.

T1-weighted MRI has an advantage in performing gross structural information, whereas T2-weighted MRI is considered the best for soft tissue contrast with biological characteristics. In this study, T2 has a lower mean volume and higher inter-observer consistency than T1. All observers were likely to focus on soft tissue contrast to reach an agreement on the T2 sequence when contoured for CTV or PTV. T2-SPAIR is a technique combining the fat selectivity of chemical shift-selective saturation and the inversion radiofrequency pulse of short-tau inversion recovery [22]. Thus, T2-SPAIR has superb contrast compared with T2 in distinguishing between tumor and normal tissues. However, for the evaluation of mean volume, CTV generated from SPAIR is 29.16 ± 12.51, which was larger than CTV-T2 (27.73 ± 13.06) and was comparable with CTV-DWI (29.25 ± 12.59). The inter-observer variety was 0.74 of DC and 0.58 of CI for CTV-SPAIR. It was 0.88 of DC and 0.78 of CI for PTV-SPAIR. Researchers reported that T2-SPAIR presented sensitivity, which is a prominent drawback, especially in highly susceptible regions such as geometric anatomical regions and air-tissue interface, resulting in heterogeneous fat suppression [23]. Heterogeneous fat suppression increases the uncertainty of boundaries for tumor and normal breast tissues, and it did not reflect the obviously improved accuracy of the target volume delineation compared with T2. Except for post-CT, the target volume outlined by all observers for the DWI was the largest among the pre-MRI sequences studied, with a mean volume of 29.25 ± 12.59. The inter-observer consistency was relatively low. The lowest CI was T1. The meta-analysis for DWI detected the mobility of water molecules diffusing in breast tissues with a calculated sensitivity of 0.84 (0.82–0.87) and a specificity of 79 (75–82) [24]. The most widely used clinical application of DWI is an adjunct sequence for conventional contrast-enhanced breast MRI [25]. The role of DWI in enhancing the accuracy of clinical target delineation is not satisfactory and requires improvement and further research. All observers were found to be most concordant with the sdyn-THRIVE sequence, followed by e-THRIVE. The e-THRIVE is a turbo field echo scan of a 3D T1 weighted with inversion recovery fat suppression; it was initially introduced for imaging of the breast, liver and other regions [26]. On the basis of e-THRIVE, the sdyn-THRIVE is an examination method that eliminates overlapping images of bone and soft tissue, thereby highlighting blood vessel images. From this method, a high-contrast image involving the high signal of a blood vessel image only in the tumor was obtained. Compared with other contouring sequences of pre-MRI that rely on tissue contrast, sdyn-THRIVE sequence more intuitively shows the outline of the tumor. The values of the conformity parameters of sdyn-THRIVE sequence, i.e., DC-CTV of 0.86 and CI-CTV of 0.74 and DC-PTV of 0.93 and CI-PTV of 0.87, were the largest among all pre-MRI sequences fused to CT. sdyn-THRIVE has a major advantage with pre-MRI for breast RT planning and can better define the location of the tumor compared with T1, T2, T2W-SPAIR, DWI and dyn-eTHRIVE. In the comparison of pre-MRI sequences, only sdyn-eTHRIVE has prominent statistical differences with all other sequences and can be expected to promote the status of target volume delineation for breast cancer patients.

This study has several limitations. One is the limited sample size. Another is the fact that the true volume and extended margin of tumor in the BCS were not clearly defined by pathological confirmation. MRI underestimates and overestimates tumor size in the ranges of 10–20 and 10–50%, respectively [27–29]. This indistinct difference between GTV defined by pre-MRI-CT fusion and pathologic resection volume cannot be clarified and studied. Other limitations of this study included the uncertainty in the rigid-registration of post-CT and pre-MRI datasets because of the nonrigid nature of breast shape. Moreover, in the time from BCS to post-CT, the instability of SC and the deformation of breast could increase the inconsistency of co-registration. The device for pre-MRI and CT positioning also resulted in registration deviation. Deformable image registration (DIR) may be an ideal tool considering the volume loss and breast changes after lumpectomy. However, DIR for multimodality image is generally not reliable and inaccurate with DIR tools [6]. The nonrigid nature of breast shape may pose an additional challenge to the use of DIR. The rigid-body registration has been used routinely in the clinic. The use of clips, the lump and various anatomic features, especially the mammary glands, minimised the registration uncertainty.

## Conclusion

This study showed important insights into the relative importance of in pre-MRI and post-CT fusion. MRI helps reduce implant/treatment volumes and potentially guides treatment planning. Pre-MRI provides a more precise definition of the TB with observers showing a smaller inter-observer variability than CT. Smaller CTV-sdyn-THRIVE and PTV-sdyn-THRIVE are highly desirable for reducing treatment volume and lead to the definition of the TB delineation as accurately as possible. Pre-MRI, especially the sdyn-eTHRIVE sequence, helps reduce treatment volumes by improving the accuracy of TB delineation of the neoadjuvant RT of breast cancer.

## Data Availability

The datasets used and analysed during the current study are available from the corresponding author on reasonable request.

## Abbreviations

TB: Tumor bed
MRI: Magnetic resonance imaging
RT: Radiotherapy
CT: Computed tomography
pre-MRI: Preoperative magnetic resonance imaging
post-CT: Postoperative computed tomography
US: Ultrasound
BCS: Breast conserving surgery
DWI: Diffusion-weighted imaging
T2W-SPAIR: T2-weighted-spectral-attenuated inversion recovery
dyn-eTHRIVE: Dynamic-enhanced TI high-resolution isotropic volume excitation
sdyn-eTHRIVE: Subtraction of dynamic-enhanced TI high-resolution isotropic volume excitation
GTV: Gross target volume
CTV: Clinical target volume
PTV: Planning target volume
CI: Consistence index
DC: Dice coefficient
CVS: Cavity visualization score
DIR: Deformable image registration
